# Sex Differences in Gross Motor Competence in Italian Children Aged 3–11 Years: A Large-Scale Cross-Sectional Study

**DOI:** 10.3390/jfmk10010061

**Published:** 2025-02-10

**Authors:** Cristiana D’Anna, Fabio Carlevaro, Francesca Magno, Roberto Vagnetti, Pierpaolo Limone, Daniele Magistro

**Affiliations:** 1 Department of Education and Sport Sciences, Pegaso University, Centro Direzionale, Isola F2, 80143 Naples, Italy; pierpaolo.limone@unipegaso.it; 2 Asti Higher Studies University Pole, University of Turin, p. Fabrizio de André, 14100 Asti, Italy; fabio.carlevaro@unito.it (F.C.); francesca.magno@unito.it (F.M.); 3 Department of Life Sciences and Systems Biology, University of Turin, V. Giuseppe Verdi, 8, 10124 Turin, Italy; 4 Department of Sport Science, School of Science and Technology, Nottingham Trent University, 50 Shakespeare St, Nottingham NG1 4FQ, UK; roberto.vagnetti@ntu.ac.uk (R.V.); daniele.magistro@ntu.ac.uk (D.M.)

**Keywords:** children, gross motor development, motor competence, sex

## Abstract

**Background/Objectives**: In recent years, there has been a significant increase in studies examining motor learning during preschool age and the early years of primary school. This study aimed to investigate sex differences in gross motor competence among Italian children aged 3–11 years. **Methods**: A convenience sample of 8500 children (mean age = 8.37 years, SD = 1.98; 50% female) was included in this cross-sectional study. Gross motor skills were assessed using the Italian version of the Test of Gross Motor Development–3, which evaluates locomotion and ball control skills. A Linear Mixed Model was applied to examine the interaction between sex and age, with school included as a random intercept and BMI as a covariate. **Results**: The results revealed a consistent trend of boys achieving significantly higher total scores for global motor competence (*p* < 0.001) across all age groups, except at age 11. Boys also demonstrated superior performance in ball control skills (*p* < 0.005) at all ages. In contrast, no significant differences were observed for locomotion skills overall. However, girls outperform boys in locomotor skills at ages 6, 7, and 8 (*p* < 0.001), with this trend disappearing by age 9. **Conclusions**: These findings highlight important sex-related differences in gross motor development during childhood, influenced by both biological and environmental factors. The results underscore the need for targeted interventions in educational settings to provide equitable opportunities for motor skill development, particularly for girls. Enhancing the quality of physical education and addressing gender disparities can support the acquisition of essential motor skills and promote lifelong physical activity.

## 1. Introduction

The developmental period represents a critical phase during which children acquire foundational motor skills essential for the subsequent expansion and qualitative enhancement of all future motor abilities, both complex and simple [1,2]. These motor skill levels play a crucial role in both the current and future well-being of children, affecting their engagement in physical activities [3] and contributing to health, sustained active lifestyles, and overall quality of life over time [1,4,5,6].

Models of human development [2,7,8] describe motor development as a process that typically follows fundamental stages (milestones) in a predictable sequence across the population. However, there is considerable individual variability in how these stages are reached. While children generally acquire similar motor skills, observing an individual child may reveal that their motor development is more or less advanced compared to another child nearby. This highlights the paradox between the universal progression of motor development and the uniqueness of individual differences [9].

The concepts of motor development and motor competence are deeply interconnected with other aspects of a person’s development, influencing cognitive and social growth [4,10,11,12,13,14] as well as academic performance [15,16,17,18].

In recent years, there has been a significant increase in studies examining motor learning during developmental stages, particularly in relation to cognitive and social development. These studies highlight the importance of focusing on motor behaviour during preschool age and the early years of primary school, a critical period for acquiring adequate levels of motor competence [13]. Motor competence refers to the ability to organise and apply acquired skills to adapt to the environment and achieve specific objectives. It reflects an individual’s performance across diverse situations and contexts and results from implicit mechanisms and processes, including movement quality, motor coordination, and motor control [19,20,21]. Previous studies have shown that improvements in controlling the body in space and time, including object manipulation during purposeful movements, result not only from natural maturation but also from social and physical environmental factors that stimulate and support learning [22]. Understanding sex differences in motor development in relation to environmental variables is particularly important. Indeed, sex significantly influences motor skill development [23], as males and females often receive different stimuli due to genetic predispositions and environmental factors [24,25]. Males are typically encouraged to engage in activities that develop strength and power, whereas females are more often provided with opportunities to practise coordination and flexibility [23,24,26]. This disparity in motor encouragement likely contributes to males being more physically active than females in daily life [27,28] and highlights the need for improved environmental support—whether at school, within families, or in the broader community [28].

The family environment plays a critical role in motor development. Parents, often the first playmates, provide encouragement and stimuli that can be influenced by sex, thereby shaping the development of motor competence [29] (Dinkel & Snyder, 2020). Studies grounded in social learning theory, particularly following Bandura (1977) [30], have investigated the combined impact of biological and sociocultural factors on acquiring fundamental motor skills (FMSs). These studies attribute sex differences in FMS performance to varying expectations and opportunities provided to children [29]. Additionally, self-determination theory suggests that intrinsic motivation is shaped by gender-specific support and encouragement [31,32]. Cultural factors significantly influence motor and sports participation, affecting the choice of activities (e.g., dance, football, gymnastics), as well as the quantity, quality, and context of practice. These cultural influences inevitably impact the acquisition and level of motor development.

Motor competence during developmental age is closely linked to gross motor development; however, studies examining sex differences in gross motor development have produced varied and sometimes contradictory findings. For example, an Indian study by Shivaraju (2024) [33] using the Test for Gross Motor Development (TGMD-2) [34] found that locomotor skills were comparable between boys and girls. In contrast, boys demonstrated significantly better ball control skills than girls, indicating potential sex-specific differences in overall motor development. This pattern of superior manipulative skills in males is consistent with findings from other studies. However, results regarding locomotor skills differ; for instance, Veldman et al. (2018) [16] reported that females outperform males in locomotor tasks, underscoring inconsistencies in sex-related motor performance across studies.

Barnett et al. (2009) [25], in their meta-analysis, attributed the higher manipulative skills observed in males to a combination of biological and social factors, such as differences in physical strength, hormonal influences, and sex-specific play patterns that emphasise these skills from an early age [35]. However, their findings showed no significant sex differences in locomotor skills, with males and females performing similarly in activities like running and jumping [4]. Similarly, a recent study on the Brazilian population found no significant sex differences in either subtest of the Test for Gross Motor Development TGMD-3 [36]. The strategies used to approach motor tasks and responses to challenges were comparable between sexes. However, this study revealed that the changes in motor learning differ significantly between age groups. Both locomotor and ball control skills showed greater improvement during the 6–10-year age range compared to the 3–5-year period [37]. These findings highlight the growing body of research on motor development during childhood and underscore the importance of understanding how fundamental motor skills evolve over time. In particular, there is a need to explore how differences in motor competence vary with age and sex [38]. Indeed, understanding motor behaviour is essential for designing effective learning environments that address the needs of all students [31]. Such environments must be adaptable to provide personalised support, ensuring that educators can tailor their approaches to optimise each child’s motor skill development.

This study aims to investigate sex differences in gross motor development during childhood by evaluating the interaction between sex and age in the 3–11-year age range. The focus is on identifying differences in motor development between males and females within the same age groups.

## 2. Materials and Methods

### 2.1. Participants

A convenience sample of 8500 children aged 3 years and 0 months to 11 years and 11 months (mean age = 8.37, SD = 1.98, 50% females) participated in this study, which is part of the longitudinal project *Benessere in Gioco* (BiG) [39,40,41]. The *Benessere in Gioco (BiG)* project, designed to analyse and understand motor and behavioural development in children, was conducted in north-western Italy and involved 31 public schools across multiple cities and towns, comprising a total of 340 classes. The schools were not randomly selected but were those that agreed to participate in the project. Invitations were extended to public schools throughout the region, and participation was determined based on the schools’ willingness to collaborate and their capacity to accommodate the study within their schedules. All participating schools operated under similar educational conditions, providing a standardised context for the assessments. Written informed consent was obtained from parents or guardians, along with assent from all participants. This study was approved by the ethics committee of the University of Turin (ID 100949).

The sample was stratified into 17 age groups: 16 groups spanning 6-month intervals and 1 group covering children aged 11 years and 0 months to 11 years and 11 months. Each group included 250 boys and 250 girls. Differently from the USA version, the Italian standardisation included children older than 11 years to encompass all primary school students. This approach also provides a reference score for evaluating gross motor skills at the transition to lower secondary school.

### 2.2. Tool

The Italian version of TGMD-3 [42] was used to evaluate gross motor development. This tool assesses fundamental movement skills, divided into two domains: locomotion skills and ball control skills. Specifically, the TGMD-3 evaluates how children manage and control whole-body movements in space and time. The test emphasises the qualitative aspects of movement rather than the outcomes of actions or performances. It systematically observes motor behaviour across 13 fundamental movement skills.

Gross motor skills were assessed based on the qualitative criteria for each TGMD-3 item as follows: every criterion was scored based on whether it was fulfilled (score awarded = 1) or not (score awarded = 0). Two trials were performed for each item, and the total score for each item was given by the sum of all the performance criteria scores in both trials. The sum of performance criteria scores from both trials was used to calculate the scores for the locomotor and ball control skill subtests, as well as for the overall TGMD-3 scores. Accordingly, the maximum score that a participant could obtain was 100 for their overall gross motor performance, 46 for the locomotor subtest, and 54 for the ball skill subtest.

The TGMD-3 was used because there is no gold standard for the assessment of gross motor skills [43]. The TGMD-3 is widely used globally for assessing these skills [44,45] and has demonstrated good psychometric properties [39,40,42,46].

However, the TGMD-3 is widely used worldwide for evaluating these skills [44,45] and has demonstrated strong psychometric properties [39,40,42,46,47]. Multiple validation studies have been conducted for the TGMD-3 [48,49,50], including the development of an Italian version [42,51].

The TGMD-3 was administered by a team of 25 professionals, including 4 sports science researchers, 2 psychologists, and 19 sports science graduates. All examiners participated in a two-hour training session to ensure consistent adherence to the test protocol. The assessments were conducted in school gyms during school hours, with physical education teachers present. Two independent testers, randomly paired for each session, simultaneously observed and scored each child’s performance in real time. The agreement between the scores recorded by the two testers exceeded 95%. Cohen’s kappa (k) was calculated to assess agreement between the two observers for all TGMD-3 criteria, with values ranging from 0.8 to 1 indicating excellent or near-perfect agreement. Additionally, inter-rater reliability for the final TGMD-3 scores was evaluated using a two-way random intraclass correlation coefficient (absolute agreement), with values above 0.90 considered excellent. At the start of each testing session, one examiner provided a clear verbal explanation and demonstration of each skill to ensure consistency and understanding. Each child performed three trials: one practice trial followed by two formal trials. Only the scores from the two formal trials were recorded for evaluation. Performances were observed and assessed based on the qualitative performance criteria outlined for each TGMD-3 skill.

### 2.3. Statistical Analysis

A Linear Mixed Model (LMM) was employed to examine differences between boys and girls. First, an unconditional means model was constructed to assess how much of the total variance in the Total, Object, and Locomotor scores of the TGMD-3 was attributable to between-school variance and to determine the necessity of a multilevel model. Specifically, the need for a multilevel approach was evaluated using the intraclass correlation coefficient (ICC), with values higher than 0.30 indicating the need for a mixed-model approach to reduce type I errors [52]. If needed, a Linear Mixed Model was developed using restricted maximum likelihood estimation, considering the children’s school as a random intercept, to test the interaction between age and sex for each dependent variable, i.e., the TGMD-3 total score and the two sub-scores, controlling for the effect of BMI. The interaction between sex and age was tested. Predictive margins derived from the model were used to estimate average marginal contrasts between boys and girls within the same age groups, with contrasts adjusted using Bonferroni correction for multiple comparisons. Statistical analyses were performed with R, lme4, lmerTest, and gg effects.

## 3. Results

**Figure 1:**
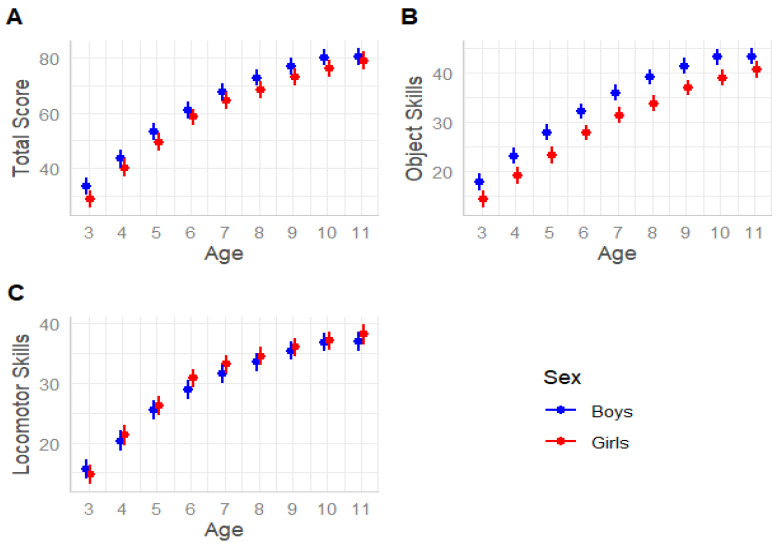
[DM1] Boys and girls scores on TGMD-3 Total (**A**), Object (**B**), and Locomotor (**C**) Scores.

Unconditional models indicated high ICC values explained by between-school variance for the TGMD-3 Total Score (ICC = 0.59), Object Score (ICC = 0.50), and Locomotor Score (ICC = 0.54), demonstrating the need for a multilevel approach. LMM results indicated an interaction between Sex and Age group in the Total Score (F(8, 16438) = 2.45, *p* = 0.011), Object Score (F(8, 16438) = 3.49, *p* < 0.001), and Locomotor Score (F(8, 16438) = 5.61, *p* < 0.001). Boys showed significantly higher Total Scores compared to girls at all ages (all *p* < 0.001) except at age 11 (*p* = 0.940), and in the Object Score at all ages (all *p* < 0.05). In the Locomotor Score, boys and girls showed similar performance overall (all *p* > 0.05), but girls had higher scores than boys at ages 6 (*p* < 0.001), 7 (*p* < 0.001), and 8 (*p* = 0.004). Results are summarised in Table 1 and Figure 1. BMI was found to be a significant covariate for the Total Score (F(1, 16445) = 547.95, *p* < 0.001), Object Score (F(1, 16445) = 286.52, *p* < 0.001), and Locomotor Score (F(1, 16445) = 461.58, *p* < 0.001), all showing negative relationships. Specifically, higher BMI was associated with lower scores in the Total Score (b = −0.62, SE = 0.002, t = −23.40, *p* < 0.001), Object Score (b = −0.29, SE = 0.001, t = −16.92, *p* < 0.001), and Locomotor Score (b = −0.34, SE = 0.001, t = −21.48, *p* < 0.001).

## 4. Discussion

The analysis of the results clearly shows a natural increase in motor competence levels as children grow older. This finding aligns with previous studies in the scientific literature, which highlight the pronounced manifestation of this trend during the 3–5-year age range [45]. When comparing average values by sex for each age from 3 to 11 years, boys consistently demonstrate higher gross motor skills with significant sex differences across all age groups, with males scoring higher than females.

This trend is also evident in the ball control skills subtest, where males showed higher scores than females across all age groups. However, the development over the years of locomotion skills shows a different pattern. Up to age 5, both sexes exhibit similar levels in locomotor skills. From around 4.5 years old, females begin to show higher average values, with significant differences (*p* < 0.001) observed in the 6- and 7-year and in the 8-year (*p* < 0.004) age group. From age 9 to 11, the average values for both sexes converge once again. These results highlight both the general progression of gross motor skills development with age and the nuanced differences in gross motor skill development between sexes. The observed sex differences in locomotor abilities during specific developmental stages, particularly at ages 6 to 8, highlight the importance of addressing sociocultural and educational factors in promoting equitable motor skill development. These findings suggest that girls may benefit from activities that emphasise locomotor skill enhancement during this critical developmental window. Conversely, tailored interventions for boys could focus on narrowing the gap in locomotor competence by incorporating activities that promote coordination and agility. Recognising the influence of societal norms and early childhood play behaviours is essential for designing inclusive physical education programmes that foster skill development equally among boys and girls. By mitigating sex-based disparities in motor skill acquisition, such initiatives can contribute to sustained engagement in physical activity and improved lifelong health outcomes.

The results align with previous studies highlighting the significant influence of sex on motor competence acquisition during childhood. Girls often demonstrate lower motor performance compared to boys [53], a disparity potentially exacerbated by the lack of encouragement for girls to participate in diverse physical activities, which negatively impacts their proficiency in locomotion and manipulation skills [54,55]. A recent study also reported higher motor competence in ball control skills among boys [38], attributing this advantage to superior rotation biomechanics involving the pelvis, torso, and shoulders during throwing. This may reflect an evolutionary perspective associating the male sex with hunting and food acquisition, further reinforced by social factors [38].

Previous studies indicate that males tend to show higher performances than females in locomotor skills such as running and jumping, as well as in object control skills like throwing and catching [56,57]. These differences are apparent from an early age and become more pronounced as children grow older [23,46]. A study [58] explored the influence of biological and environmental factors on the throwing performance of 5-year-old boys and girls. The study considered variables such as participation in activities with adults and older children. The findings revealed that boys performed better in throwing, both in terms of distance and coordination. While part of this advantage was attributed to biological factors predisposing males to superior performance, the study highlighted that, particularly from age 5 to puberty, differences in performance were largely due to environmental factors. Specifically, boys were found to have greater opportunities for practice through play and sports activities with older children or adults compared to girls [57].

Another study examining sex differences during developmental age argued that while the stages of motor development are the same for both males and females, girls tend to exhibit lower levels of global motor competence, object control, and manipulation skills compared to boys [59]. Conversely, girls perform better in fine motor skills and balance abilities [59]. These findings are consistent with other studies reporting higher performance by girls in manual dexterity [59,60] and balance [59,60,61,62] compared to boys. Meanwhile, boys demonstrate higher levels of global motor skills and greater proficiency in object control and manipulation [59,62,63]. 

This study found that higher BMI is associated with lower motor skills. Higher BMI is often associated with lower motor competence in children, meaning that children with higher BMI may face more difficulties with activities that require coordination, balance, and agility [44,64,65]. This relationship underscores the role of BMI in motor performance [66]. A child’s weight status, whether healthy or risky, could be influenced by a cycle where motor competence impacts physical activity engagement, which in turn affects BMI [1]. This positive or negative spiral emphasises the need for targeted interventions to improve both motor skills and overall health. Considering BMI in the design of early interventions is crucial not only because of its impact on motor competence but also due to its broader health implications [67]. Children classified as obese at a young age are of particular concern, as research indicates they are unlikely to achieve a normal weight solely through natural growth.

As widely discussed in the literature, the development process is shaped by the interaction between biological potential and environmental factors. Changes in an individual occur through continuous interaction with the environment, where internal and external influences do not simply accumulate but interact in complex ways. These interactions shape the child’s experiences and, consequently, the specific skills they acquire [68].

Motor competence is strongly linked to the daily movements children perform [69,70,71] and with greater opportunities for practice enhancing the development and refinement of fundamental motor skills [72,73].

Both the family environment—shaped by factors such as socioeconomic status and the opportunities it provides for growth in a supportive setting—and educational contexts, particularly schools, play pivotal roles in promoting motor development. Given that children spend a significant portion of their day in school, the educational context has a substantial influence. However, the motor training offered in some educational settings, e.g., the formal context of the school in which time allocated to motor and sports activities is currently limited and not always adequately planned, may fall short in both quantity and quality, failing to provide sufficient stimuli for adequate motor development. This is particularly critical during nursery and the early primary school years, a period of rapid and transformative development [74,75,76]. High-quality educational interventions during this time are essential for fostering motor development, supporting the acquisition of fundamental motor skills, and ensuring a smooth and effective progression from basic to more advanced stages of motor competence [73,77].

In the Italian school system, physical and sports activities are often marginalised due to various political, organisational, and educational challenges. This has led to a growing reliance on external organisations, such as recreational and sports centres, which predominantly offer specialised activities. Indeed, the Openpolis survey (2022) [78] examined the sports preferences of Italian children. Among those aged 3–10 years, swimming is one of the most popular sports, ranking first among girls (48.7% of those who participate in sports) and second among boys (39.4%), following football (43.7%). Generally, boys in this age group prefer ball sports, while girls gravitate towards activities like dance, gymnastics, and athletics. This pattern reflects a broader trend in Italy, where ball sports are favoured for boys and free-body activities for girls. These preferences align with our findings, which show girls excelling in free-body skills and boys performing better in ball-related skills. Additionally, national statistics reveal significant sex disparities in sports participation. According to the ISTAT report (2021) [79], continuous sports practice is more prevalent among boys (27.9%) than girls (19.6%), with occasional participation also slightly higher among boys (11.9% compared to 10.0% for girls). This lower participation rate among girls reduces their exposure to environmental stimuli that support motor skill development.

This study has several strengths. The large sample size of 8500 children, stratified across 17 age groups, provides a robust and representative analysis of gross motor development within the Italian context. The use of the TGMD-3, a widely validated and internationally recognised tool, ensures reliable and comparable results. The cross-sectional design, covering multiple age groups from 3 to 11 years, effectively captures age-related trends and highlights sex differences in motor competence during childhood. The use of a Linear Mixed Model and adjustments for confounding factors such as BMI further strengthen the validity of the findings. Importantly, this study offers practical recommendations for improving physical education and promoting equitable opportunities for motor skill development, particularly for girls.

However, some limitations must be acknowledged. The use of convenience sampling, despite the large size, may limit the generalisability of the findings beyond north-western Italy. While environmental factors are recognised, detailed data on influences such as socioeconomic status, parental involvement, and participation in extracurricular activities were not collected. Moreover, although examiners received standardised training, there remains a possibility of observer bias influencing the scoring process. Despite these limitations, the strengths of this study provide solid results for understanding age- and sex-related differences in gross motor development and highlight the need for targeted interventions to support motor skill acquisition in school-aged children.

## 5. Conclusions

This study showed that boys consistently achieved significantly higher total scores for global motor development than girls. This difference was highly significant across all age groups (*p* < 0.001), except at age 11. Similarly, boys scored higher in ball control skills (*p* < 0.005). In contrast, performance levels in locomotion skills were generally comparable between sex, except for the 6-, 7- (*p* < 0.001), and 8-year-old groups (*p* < 0.05), where girls outperformed boys.

The results highlighted the important need for targeted actions to address the identified differences in motor competence. First, at an organisational level, there is a need to increase the hours (currently one hour a week) dedicated to physical education within schools. Leveraging school autonomy for greater flexibility in curricular planning and forming partnerships with local sports clubs could help provide children, particularly girls, with equal opportunities to engage in diverse physical activities. Second, at a methodological and didactical level, teachers must be equipped to create inclusive and effective learning environments that foster the development of fundamental motor skills for all and each child. Adopting multidisciplinary approaches from a biopsychosocial perspective [80,81], addressing potential barriers, enhancing facilitators of motor development, and designing personalised intervention programmes are crucial steps to ensure that every child can reach their full potential.

## Figures and Tables

**Table 1:** [DM1] Estimated marginal mean (SE) of boys and girls for TGDM-3 scores with 95% confidence intervals for contrasts. * denotes a significant difference at *p* < 0.05.

Total Score	Boys	Girls	Contrast [95%CIs]	*p*-Value
3 years old	33.48 (1.60)	29.06 (1.61)	4.42 [3.06, 5.78]	<0.001 *
4 years old	43.50 (1.62)	40.37 (1.61)	3.13 [1.64, 4.62]	<0.001 *
5 years old	53.34 (1.59)	49.58 (1.60)	3.76 [2.33, 5.19]	<0.001 *
6 years old	61.06 (1.52)	58.55 (1.52)	2.50 [1.60, 3.40]	<0.001 *
7 years old	67.44 (1.52)	64.43 (1.52)	3.01 [2.12, 3.90]	<0.001 *
8 years old	72.67 (1.52)	68.26 (1.53)	4.41 [3.49, 5.33]	<0.001 *
9 years old	76.75 (1.52)	72.95 (1.53)	3.80 [2.87, 4.73]	<0.001 *
10 years old	80.00 (1.53)	76.05 (1.53)	3.95 [2.99, 4.91]	<0.001 *
11 years old	80.22 (1.59)	78.86 (1.62)	1.35, [−0.28, 2.99]	0.940
**Ball Skills Score**	**Boys**	**Girls**	**Contrast [95%CIs]**	***p*-Value**
3 years old	17.81 (0.86)	14.24 (0.87)	3.57 [2.71, 4.43]	<0.001 *
4 years old	23.10 (0.88)	19.04 (0.87)	4.06 [3.12, 5.00]	<0.001 *
5 years old	27.87 (0.86)	23.28 (0.86)	4.59 [3.69, 5.50]	<0.001 *
6 years old	32.20 (0.80)	27.78 (0.80)	4.41 [3.84, 4.98]	<0.001 *
7 years old	35.94 (0.80)	31.34 (0.80)	4.60 [4.04, 5.16]	<0.001 *
8 years old	39.17 (0.80)	33.79 (0.81)	5.39 [4.81, 5.96]	<0.001 *
9 years old	41.37 (0.81)	36.96 (0.81)	4.41 [3.83, 5.00]	<0.001 *
10 years old	43.21 (0.81)	39.01 (0.81)	4.20 [3.59, 4.81]	<0.001 *
11 years old	43.34 (0.85)	40.73 (0.88)	2.61 [1.58, 3.65]	<0.001 *
**Locomotor Skills Score**	**Boys**	**Girls**	**Contrast [95%CIs]**	***p*-Value**
3 years old	15.60	14.75	0.85 [0.05, 1.66]	00.343
4 years old	20.33	21.26	−0.93 [−1.81, −0.05]	00.344
5 years old	25.41	26.24	−0.83 [−1.68, 0.02]	00.494
6 years old	28.88	30.79	−1.91 [−2.45, −1.38]	<0.001 *
7 years old	31.53	33.11	−1.59 [−2.11, −1.06]	<0.001 *
8 years old	33.52	34.49	−0.97 [−1.51, −0.43]	0.004 *
9 years old	35.40	36.01	−0.61 [−1.16, −0.07]	0.255
10 years old	36.81	37.06	−0.25 [−0.82, 0.32]	0.999
11 years old	36.89	38.16	−1.26 [−2.23, −0.29]	0.096

## Data Availability

The data presented in this study are available upon request from the corresponding author.
